# Analysis of growth resistance mechanisms and causes in tea plants (*Camellia sinensis*) in high-pH regions of Northern China

**DOI:** 10.3389/fnut.2023.1131380

**Published:** 2023-02-28

**Authors:** Chun-Lei Li, Jing Xu, Hong-Mei Xu, Jie Liu, Long-Xiang Zhang, Zi-Kai Wang

**Affiliations:** ^1^Shandong Facility Horticulture Bioengineering Research Center/College of Agronomy, Weifang University of Science and Technology, Shouguang, China; ^2^Department of Tourism and Hotel Management, PingDingShan Vocational and Technical College, Pingdingshan, China; ^3^College of Horticultural Science and Engineering, Shandong Agricultural University, Tai'an, China

**Keywords:** tea tree, high pH, soil properties, growth resistance mechanism, elements

## Abstract

**Background:**

In tea plantations with high-pH (pH > 6.5) in Northern China, tea plants are prone to yellowing disease, albinism, and reductions in components that contribute to plant quality, which affect the scale and rate of tea plantation development in Northern China.

**Methods:**

To investigate the potential causes of these issues, *Camellia sinensis cv*. Pingyang Tezao and *Camellia sinensis cv*. Ruixue were planted in Shouguang city (a high-pH area, soil pH > 6.5) and Rizhao city (a normal-pH area, soil pH is 4.5–5.5), respectively; differences in growth morphology, pigment content, cell structure, quality-determining components, and element content of the two varieties in the two areas were analyzed.

**Results:**

The results showed that tea leaves planted in Shouguang had varying degrees of yellowing disease and albinism; the pigment content in both varieties was significantly lower when planted in Shouguang compared with Rizhao. The cell structure was severely damaged and the main quality-determining components were decreased. Nitrogen (N), phosphorus (P), potassium (K), zinc (Zn), copper (Cu) and manganese (Mn) contents in the leaves of the two tea plant varieties were significantly lower when planted in Shouguang compared with those in Rizhao; the levels of these elements in Shouguang soil were significantly higher than in Rizhao soil. Calcium (Ca) contents in Shouguang soil was 9.90 times higher than that of Rizhao soil.

**Conclusions:**

We conclude that the soil in high-pH areas hindered tea plant uptake of N, Zn, Cu, and Mn, which had a detrimental effect on chloroplasts and reductions in chlorophyll synthesis, contributing to yellowing disease and albinism. In addition, excessive calcium (Ca) in Shouguang soil was also an important contributor to these negative effects. High-pH soil hindered tea plant uptake of P and K, resulting in reductions in tea polyphenols, amino acids, and other major quality components.

## Introduction

Shandong Province is the main tea-producing area in Northern China. The tea produced in this province is considered to be of good quality, with a high aroma, strong taste, and brewing resistance, and is preferred by consumers. The high-quality nature of the tea has contributed substantially to the development of the Shandong tea industry, which, in turn, has become a significant contributor to Shandong's agricultural economy, increasing income for farmers. Tea planting in this province has gradually expanded from the coastal hilly areas and is increasing year by year.

However, the Shandong tea area is not optimal for tea growth. It is dry in winter, with low temperatures and rainfall and high soil salinity and pH. This does not support the growth of tea plants, which require warmth, humidity, and acidic soil to grow well. The optimal soil pH range for tea plants is 4.5–5.5 (1, 2); the pH in many areas of Shandong is higher than 6.5, and young tea seedlings struggle to reach maturity, resulting in yellowing disease, albinism, and other diseases (3) negatively affecting the quality of the plants. Soils in high-pH areas affect the growth of tea trees, while, in fact, high pH is the result of the interaction of chemical elements in the soil, and the real factors affecting the growth of tea trees are certain chemical elements in the soil. Therefore, the physicochemical elements in high-pH (pH > 6.5) soils are the main factors limiting the scale of tea cultivation and achieving high yield and quality tea in Shandong. There are a few relevant studies on the causes and prevention of yellowing disease, albinism, and quality degradation in tea plants grown in high-pH soil. In this study, tea plants were grown in an area with high-pH soil (Shouguang city in Shandong Province, soil pH = 6.84) and in an area with lower pH, which is more suitable for tea plant growth (Rizhao city in Shandong Province, soil pH = 4.56), and differences in the growth, morphology, and physiological and biochemical indexes and elemental content of tea plants in the two areas were analyzed. We also conducted an elemental analysis of the soils in the two areas to determine how soil properties might affect yellowing disease, albinism, and tea leaf quality. Finally, we explored foliar formula fertilization and proposed prevention measures against yellowing disease and albinism to provide a theoretical basis for the successful expansion of the tea planting area in high-pH (>6.5) soils. The ultimate aim of this study was to provide a scientific basis for expanding tea plantations in Shandong, in turn improving the agricultural economy and increasing the income of farmers in this region.

## Materials and methods

### Plant materials

The tea seedling varieties used in this study were *Camellia sinensis cv*. Pingyang Tezao and *Camellia sinensis cv*. Ruixue, which were 1.5 years of age. Plants were purchased from Jufeng town, Rizhao city, Shandong Province, and planted in April 2018 at experimental bases in Weifang University of Science and Technology (Shouguang city, SG) in Pingjia village, Jufeng town, Rizhao city (Rizhao city, RZ). Plants were all managed using the same techniques. Plant leaves at the third and fourth leaf position were harvested from plants in October 2018. The experimental site at the Weifang University of Science and Technology had high soil pH (6.84) with a brown soil type; the experimental site at Jufeng town, Rizhao city, had a lower soil pH that is optimal for tea growth (4.56) and a brown loam soil type.

### Determination of chlorophyll and carotenoid content

The leaf pigment content was determined using acetone extraction spectrophotometry, as previously described (4).

### Ultrastructural observation of chloroplasts

The fourth functional leaf of the new annual leaves of tea plants was selected and leaf cells were observed using a transmission microscope according to previously published methods (5).

### Determination of soil properties

Soil samples were taken from Rizhao and Shouguang experimental sites, and five points of soil (0–20 cm) were taken in the shape of an S and mixed well for use. The pH of a mixture containing soil and water in a ratio of 1:1 was measured using a pH meter (Orion 3 STAR, Thermo Fisher Scientific, Waltham, MA, USA). Soil organic matter (SOM) was measured using the potassium dichromate volumetric method, and total nitrogen was measured using a C/N elemental analyzer (Vario Max Elementar, Germany). Effective state elements were extracted from a water-to-soil mixture in a ratio of 10:1 using an M3 leaching agent, shaken for 5 min, and measured using an iCAP6300 inductively coupled plasma spectrometer (Thermoelectric Corporation, USA) (6).

### Soil nutrient grading standard of tea plantations

Table 1 shows the soil nutrient grading standard of tea plantations (7).

**Table 1:** Classification standards of soil properties/nutrients in the tea garden (14) (total nitrogen and soil organic matter units are g kg^−1^, others are mg kg^−1^).

**Property/nutrient**		**Classification of soil nutrient status**	
	**I**	**II**	**III**
pH	<4.5	4.5–5.5	>5.5
Soil organic matter	>20	15–20	<15
Total nitrogen	>1.0	0.8–1.0	<0.8
Available phosphorous	>20	5.0–20	<5.0
Available potassium	>120	80–120	<80
Available copper	>2.0	1.0–2.0	<1.0
Available manganese	>30	15–30	<15
Available zinc	>2.0	0.5–2.0	<0.5

### Elemental content of tea leaves

The levels of chemical elements were determined by inductively coupled plasma emission spectroscopy (ICP-OES) (8).

### Quality-determining component analysis

The tea polyphenol content was determined using the ferrous tartrate colorimetric method (9); the free amino acid content was determined using the ninhydrin colorimetric method (10); the soluble sugar content was determined using the anthrone colorimetric method (11); the protein content was determined using the Coomassie Brilliant Blue method (11); and the water-soluble leachate content was determined using the full amount method (11).

### Foliar spray fertilization

After planting Shouguang tea seedlings (Pingyang Tezao and Ruixue), according to the results of the single-factor experiment of foliar spray fertilization, a set of optimized programs of foliar formula fertilization to improve the yellowing disease and albinism of tea leaves, 0.8% urea + 0.8% potassium dihydrogen phosphate + 0.1% manganese sulfate + 0.01% copper sulfate + 0.01% zinc sulfate, was sprayed every 15 days for 6 months, and control sprayed with water.

### Data analysis

Microsoft Excel 2010 was used for data analysis and graph production, and SAS software was used for the LSD significance test (LSD method, *p* < 0.05).

## Results

### Morphological characteristics of tea seedlings

**Figure 1:**
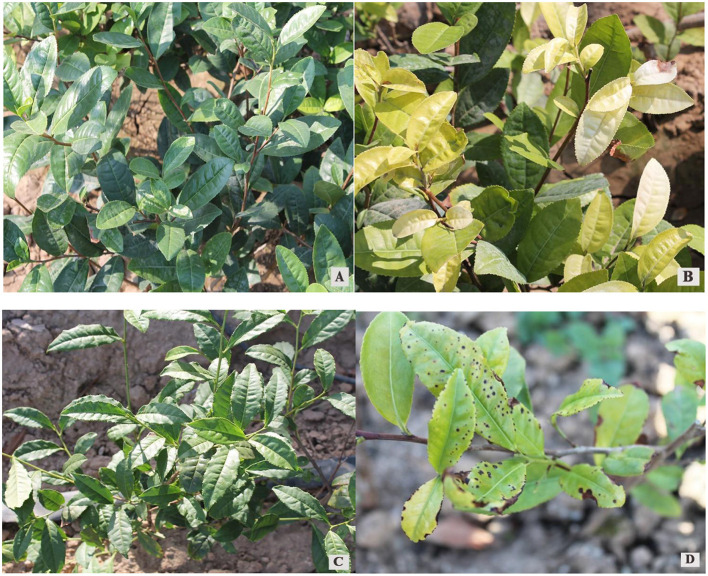
Growth patterns of Pingyang Tezao and Ruixue grown in Rizhao city and Shouguang city. **(A, C)** Pingyang Tezao and Ruixue grown in the Rizhao city tea area, respectively. **(B, D)** Pingyang Tezao and Ruixue, respectively, grown in Shouguang city.

Pingyang Tezao and Ruixue planted in Rizhao grew well, with green leaves (Figures 1A, C). The Pingyang Tezao planted in Shouguang had yellowing disease and albinism (Figure 1B), and the Ruixue planted in Shouguang had yellowing disease, with withered leaf margins and the development of black spots on the leaves (Figure 1D).

### Chlorophyll and carotenoid contents of pingyang tezao and ruixue tea plants in Rizhao city and Shouguang city

The chlorophyll and carotenoid contents of Pingyang Tezao and Ruixue grown in Rizhao and Shouguang differed greatly (Table 2). Chlorophyll a, chlorophyll b, total chlorophyll, and carotenoid contents of Rizhao-grown Pingyang Tezao and Ruixue were significantly higher and chlorophyll a/b was significantly lower than those grown in Shouguang. Specifically, chlorophyll a, chlorophyll b, total chlorophyll, and carotenoid contents of Pingyang Tezao grown in Rizhao were 84.5%, 110.7%, 91.1%, and 61.3% higher than those grown in Shouguang, respectively, while chlorophyll a, chlorophyll b, total chlorophyll, and carotenoids of Ruixue grown in Rizhao were 84.5, 104.9, 90.3, and 90.0% higher than those grown in Shouguang, indicating that the chlorophyll synthesis in Pingyang Tezao and Ruixue was blocked when cultivated in Shouguang soil.

**Table 2:** Pigment content of tea leaves in Rizhao city and Shouguang city.

**Plantation**	**Variety**	**Chl a content (mg kg^−1^FW)**	**Chl b content (mg kg^−1^FW)**	**Tol.Chl content (mg kg^−1^FW)**	**Chl a/b**	**Carotenoid content (mg kg^−1^FW)**
RZ	PYTZ	1.55 ± 0.024^a^	0.59 ± 0.021^a^	2.14 ± 0.016^a^	2.68 ± 0.022^b^	0.50 ± 0.019^a^
SG		0.84 ± 0.016^b^	0.28 ± 0.027^b^	1.12 ± 0.087^b^	2.99 ± 0.021^a^	0.31 ± 0.007^b^
RZ	RX	1.90 ± 0.032^a^	0.84 ± 0.031^a^	2.74 ± 0.071^a^	2.27 ± 0.018^b^	0.76 ± 0.023^a^
SG		1.03 ± 0.013^b^	0.41 ± 0.015^b^	1.44 ± 0.030^b^	2.51 ± 0.022^a^	0.40 ± 0.015^b^

Superscript letters a and b indicate significant differences between the two plantations (p <0.05).

RZ, Rizhao; SG, Shouguang;, PYTZ, Pingyang Tezao; RX, Ruixue.

### Ultrastructural observation of tea leaves

**Figure 2:**
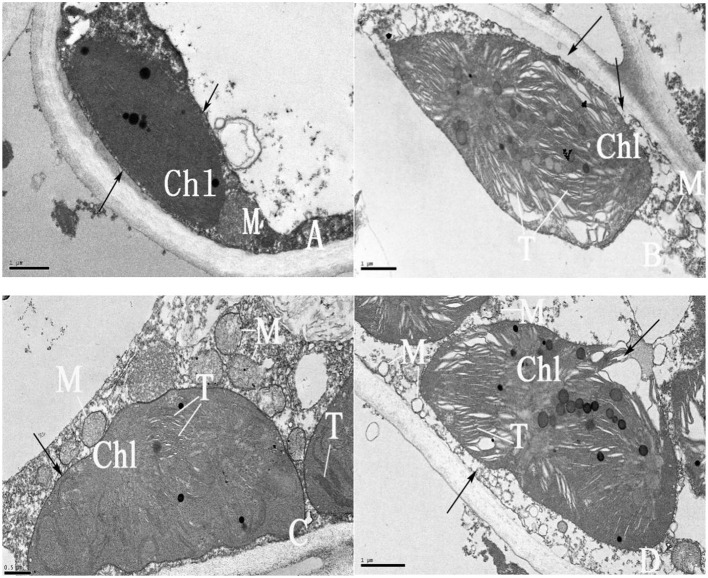
Ultrastructure of Pingyang Tezao and Ruixue leaves grown in Rizhao city and Shouguang city. **(A, C)** Pingyang Tezao and Ruixue, respectively, grown in the Rizhao city tea area. **(B, D)** Pingyang Tezao and Ruixue leaves, respectively, grown in the Shouguang city tea area. Scale bars in **A, B**, and **D** = 1 μm; scale bar in **C** = 0.5 μm; Chl, chloroplast; M, mitochondria; T, thylakoid.

Chloroplast structures in the leaves of Pingyang Tezao and Ruixue plants grown in Rizhao and Shouguang are shown in Figure 2. Chloroplast structures of Pingyang Tezao and Ruixue grown in Rizhao were complete and elliptical (Figures 2A, C), with clearly observable chloroplast envelopes and cell membranes (Figures 2A, C, black arrow). Mitochondria were intact, with complete membrane structures and clear cristae (Figures 2A, C, black arrow). In contrast, chloroplasts of Pingyang Tezao and Ruixue leaves from plants grown in Shouguang were structurally disordered, with distorted vesicle lamellae, severe separation of the plastid wall, dissolved cell membrane (Figures 2B, D, black arrow), and cavitated mitochondria. Ruixue chloroplasts were broken by the periplasm (Figure 2D, black arrow). Given that chloroplasts are the site of chlorophyll formation, disruption of their structure results in decreased chlorophyll content.

### Analysis of the quality-determining components of tea leaves

The levels of quality-determining components of tea plants in Rizhao and Shouguang are shown in Table 3. Polyphenols, amino acids, and soluble extracts of the two varieties cultivated in Rizhao were significantly higher than those cultivated in Shouguang. Polyphenols in the leaves of Pingyang Tezao and Ruixue grown in Rizhao were 89.6 and 74.8% higher than their equivalents grown in Shouguang, respectively; amino acids in the leaves were 22.3 and 62.3% higher in plants grown in Rizhao compared with Shouguang, indicating that Shouguang soil reduced the synthesis of these major components of tea. Soluble sugar and protein contents were both higher in Shouguang planting than in Rizhao (Table 3).

**Table 3:** Analysis of quality components of Pingyang Tezao and Ruixue grown in Rizhao city and Shouguang city (%).

**Plantation**	**Variety**	**Polyphenols**	**Amino acids**	**Soluble-sugar**	**Protein**	**Water soluble extracts**
RZ	PYTZ	22.58 ± 0.14^a^	3.68 ± 0.08^a^	4.61 ± 0.05^b^	5.56 ± 0.10^a^	38.33 ± 0.29^a^
SG		11.91 ± 0.02^b^	3.01 ± 0.03^b^	9.28 ± 0.01^a^	5.73 ± 0.12^a^	33.60 ± 0.19^b^
RZ	RX	28.17 ± 0.22^a^	3.88 ± 0.03^a^	3.26 ± 0.21^b^	5.29 ± 0.10^a^	40.56 ± 0.34^a^
SG		16.12 ± 0.13^b^	2.39 ± 0.01^b^	8.41 ± 0.05^a^	5.57 ± 0.10^a^	34.86 ± 0.26^b^

RZ, Rizhao; SG, Shouguang; PYTZ, Pingyang Tezao; RX, Ruixue.

Superscript letters a and b indicate significant differences between the two plantations (*p* <0.05).

### Elemental content of tea leaves

The elemental contents of tea leaves in the two plantations differed greatly, as shown in Table 4. N, P, K, Mn, Zn, and Cu in the leaves of the two varieties planted in Rizhao were significantly higher than in Shouguang. Pingyang Tezao and Ruixue plants grown in Rizhao had a leaf N content that was 35.6 and 59.1% higher, respectively, compared with plants grown in Shouguang; leaf P was 191.7 and 275.7% higher; leaf K was 33.5 and 30.3% higher; leaf Mn was 270.7 and 628.0% higher; leaf Zn was 138.7 and 303.2% higher; and leaf Cu was 379.3 and 303.2% higher. In contrast, Ca, Mg, and B levels in the leaves of the two varieties planted in Rizhao were significantly lower than in Shouguang. Pingyang Tezao and Ruixue plants grown in Rizhao had a leaf Ca content that was 82. 1 and 82.8% lower in Rizhao, respectively, compared with plants grown in Shouguang; leaf Mg content was 43.3 and 38.3% lower; and leaf B content was lower at 39.2 and 17.4% lower. S and Fe contents of the two varieties did not differ significantly between the two locations.

**Table 4:** Leaf elemental content analysis in Pingyang Tezao and Ruixue grown in Rizhao city and Shouguang city (Zn, Cu, and B units are mg kg^−1^; all other units are g kg^−1^).

**Plantation**	**Variety**	**N**	**P**	**K**	**Ca**	**Mg**	**S**	**Fe**	**Mn**	**Zn**	**Cu**	**B**
RZ	PYTZ	53.60 ± 0.51^a^	4.58 ± 0.13^a^	18.00 ± 0.14^a^	4.07 ± 1.12^b^	2.02 ± 0.06^a^	2.32 ± 0.36^a^	0.41 ± 0.15^a^	1.52 ± 0.31^a^	59.21 ± 3.15^a^	24.78 ± 0.82^a^	20.68 ± 1.30^b^
SG		39.36 ± 0.25^b^	1.57 ± 0.07^b^	13.48 ± 0.11^b^	22.74 ± 0.53^a^	3.56 ± 0.32^b^	2.22 ± 0.19^a^	0.42 ± 0.26^a^	0.41 ± 0.07^b^	24.81 ± 2.37^b^	5.17 ± 0.24^b^	34.00 ± 3.51^a^
RZ	RX	58.85 ± 0.17^a^	5.41 ± 0.17^a^	20.01 ± 0.18^a^	3.56 ± 0.27^b^	1.84 ± 0.14^b^	2.73 ± 0.11^a^	0.45 ± 0.31^a^	1.82 ± 0.55^b^	54.69 ± 3.82^a^	21.33 ± 1.0^a^	34.14 ± 2.94^b^
SG		36.99 ± 0.31^b^	1.44 ± 0.11^b^	15.36 ± 0.26^b^	20.64 ± 0.95^a^	2.98 ± 0.31^a^	2.35 ± 0.13^a^	0.49 ± 0.23^a^	0.25 ± 0.13^a^	29.29 ± 1.51^b^	5.29 ± 0.33^b^	41.31 ± 0.67^a^

RZ, Rizhao; SG, Shouguang; PYTZ, Pingyang Tezao; RX, Ruixue.

Superscript letters a and b indicate significant differences between the two plantations (*p* <0.05).

Chloroplasts deficient in N, sulfur (S), magnesium (Mg), iron (Fe), Mn, Cu, and Zn become chlorotic. This is because N and Mg comprise chlorophyll, Fe is a component of iron–sulfur protein in the chloroplast photosynthetic electron transfer system of chloroplasts, and Mn, Cu, and Zn are activators of certain enzymes in the chlorophyll formation process. All of these functions are closely related to the formation of chlorophyll or chloroplasts. In the absence of Mn, vesicles, the basic structural unit of chloroplasts, cannot form lamellae, and with Zn deficiency, the inner membrane system of chloroplasts is disrupted (12–14). The two varieties grown in Shouguang had serious damage to the chloroplast structure (Figure 2), distortion and breakage of the cystoid, and destruction of the chloroplast membrane and cell membrane, resulting in the reduction of chlorophyll content. It is likely that the yellowing and albinism of tea plants in high-pH areas were caused by the element deficiencies we observed. In addition, a high concentration of Ca damages chloroplast lamellae and decreases chlorophyll content (15); Ca may also be an important cause of the yellowing and whitening of tea plants in high-pH areas.

### Soil analysis in Rizhao and Shouguang

The properties of soils in Rizhao and Shouguang differed greatly, as shown in Table 5. The pH of the soil in the Rizhao tea area was 4.56, which was within the suitable range for tea plant growth (pH 4.5–5.5), and the pH of Shouguang soil was 6.84, which was outside of this range (considered high pH). Referring to soil nutrient grading standards of tea plantations (Table 1) (7), the total N content and SOM content of Shouguang soil reached the grade I standard, and the total N content and SOM content of Rizhao soil reached the grade II standard. The total N content of Shouguang soil was 1.83 times higher than that of Rizhao soil, and the SOM content in Shouguang was 2.23 times higher than in Rizhao soil. The effective Zn, effective Cu, and effective Mn in both Shouguang and Rizhao soils reached the grade I standard, while the effective Zn and effective Cu in Shouguang soil were significantly higher than those in Rizhao soil, and the effective Mn of Shouguang soil was significantly lower than in Rizhao soil.

**Table 5:** Soil composition analysis of Rizhao city and Shouguang city tea plantations (total nitrogen and organic matter units are g kg^−1^, all other units are mg kg^−1^).

**Plantation**	**pH**	**Total N**	**Organic matter**	**Available P**	**Available K**	**Available Ca**	**Available Mg**	**Available Fe**	**Available Zn**	**Available Cu**	**Available Mn**
RZ	4.56 ± 0.02^a^	0.83 ± 0.01^b^	14.5 ± 0.22^b^	222.3 ± 5.2^a^	135.8 ± 3.1^a^	660.1 ± 12.3^b^	132.9 ± 5.5^b^	340.7 ± 8.5^a^	5.37 ± 0.31^b^	2.62 ± 0.09^b^	171.3 ± 5.7^a^
SG	6.84 ± 0.05^b^	1.52 ± 0.03^a^	32.4 ± 0.71^a^	152.3 ± 3.6^b^	115.1 ± 2.8^b^	6543.3 ± 70.4^a^	570.0 ± 21.4^a^	290.1 ± 10.3^b^	6.42 ± 0.39^a^	2.96 ± 0.05^a^	67.1 ± 2.3^b^

RZ, Rizhao; SG, Shouguang. Superscript letters a and b indicate significant differences between the two plantations (*p* <0.05).

According to the soil nutrient grading standards (Table 1) (7), the effective P of both Rizhao and Shouguang soils reached class I standard, the effective K of Shouguang soil reached class II standard, and the effective K of Rizhao soil reached class I standard. The content of effective P and effective K in Shouguang soil was significantly lower than that in Rizhao soil, resulting in significantly lower P and K in the leaves of both varieties grown in Shouguang compared with Rizhao (Table 4). P facilitates the formation of polyphenols in tea leaves, and P deficiency affects the conversion of sugars into polyphenols, which leads to the accumulation of sugars in leaves (16). As shown in Table 3, the soluble sugar contents of the two tea varieties grown in Shouguang were more than two times that of the tea grown in Rizhao. As shown in Tables 4, 5, the effective K levels in Rizhao soil and Rizhao-grown tea leaves are both higher than those in Shouguang soil and Shouguang-grown tea leaves. The K content in tea leaves is positively correlated with total amino acid levels, and K^+^ ions regulate stomatal movement and promote photosynthesis, thus facilitating the formation of sugars and polyphenols, which likely explains the high tea polyphenol and amino acid contents in Rizhao-grown tea leaves compared with those grown in Shouguang.

### Effect of foliar spraying of formulated fertilizer on the control of yellowing disease and albinism

**Figure 3:**
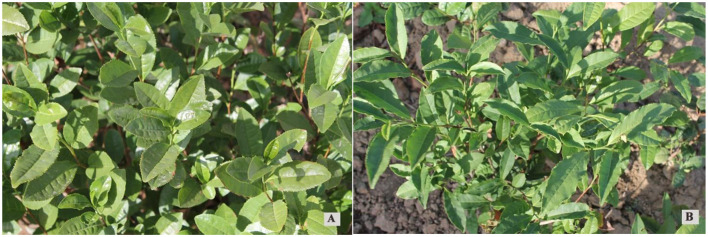
Growth pattern of Pingyang Tezao and Ruixue after foliar spray fertilization. **(A)** Pingyang Tezao; **(B)** Ruixue.

The elemental content of tea leaves before and after foliar spray fertilization on Pingyang Tezao and Ruixue planted in Shouguang is shown in Table 6. The elemental contents of N, P, K, S, Mn, Zn, and Cu of the leaves were significantly higher after fertilization compared with a non-fertilized control, while Ca, Mg, Fe, and B contents were not significantly different. Chlorophyll a, chlorophyll b, total chlorophyll, and carotenoid contents of Pingyang Tezao and Ruixue were significantly higher after fertilization than in the non-fertilized control plants (Table 7). After foliar spray fertilization, Pingyang Tezao and Ruixue plants had greener leaves and good growth, and the preventative effect on yellowing diseases and albinism was clear (Figure 3). This suggests that higher soil pH reduced the uptake of N, Zn, Cu, and Mn by tea plants, resulting in the destruction of chloroplast structure and decreased chlorophyll content, resulting in yellowing and albinism.

**Table 6:** Leaf elemental content analysis of Pingyang Tezao and Ruixue before and after fertilizer spraying (Zn, Cu, and B units are mg kg^−1^, all other units are g kg^−1^).

**Variety**	**N**	**P**	**K**	**Ca**	**Mg**	**S**	**Fe**	**Mn**	**Zn**	**Cu**	**B**
**PYTZ**	33.27 ± 0.85^b^	1.43 ± 0.19^b^	12.23 ± 0.17^b^	21.83 ± 0.29^b^	3.02 ± 0.11^a^	2.14 ± 0.14^b^	0.40 ± 0.10^a^	0.45 ± 0.07^b^	26.72 ± 0.54^b^	5.65 ± 0.33^b^	32.67 ± 1.09^a^
	48.82 ± 2.33^a^	3.51 ± 0.34^a^	16.52 ± 0.44^a^	23.75 ± 0.38^a^	3.30 ± 0.24^a^	3.47 ± 0.03^a^	0.43 ± 0.03^a^	1.02 ± 0.05^a^	42.33 ± 2.21^a^	18.34 ± 0.47^a^	34.53 ± 3.27^a^
**RX**	35.23 ± 1.71^b^	1.74 ± 0.08^b^	16.69 ± 0.72^b^	21.42 ± 0.92^a^	3.13 ± 0.30^a^	2.18 ± 0.20^b^	0.47 ± 0.08^a^	0.29 ± 0.03^b^	31.75 ± 1.03^b^	5.52 ± 0.62^b^	35.27 ± 2.34^a^
	52.67 ± 0.52^a^	4.36 ± 0.51^a^	18.57 ± 1.37^a^	22.57 ± 1.57^a^	2.84 ± 0.64^a^	3.63 ± 0.36^a^	0.44 ± 0.19^a^	0.72 ± 0.03^a^	44.89 ± 0.84^a^	16.57 ± 2.14^a^	36.93 ± 1.57^a^

PYTZ, Pingyang Tezao; RX, Ruixue. Superscript letters a and b indicate significant differences between before and after fertilization of the same variety (*p* <0.05). The 1st and 2nd line of the same variety is the control and experimental group separately.

**Table 7:** Analysis of pigment content of tea leaves before and after fertilizer spraying.

**Variety**	**Chl a content (mg kg^−1^ FW)**	**Chl b content (mg kg^−1^ FW)**	**Tol Chl content (mg kg^−1^ FW)**	**Chl a/b**	**Carotenoid content (mg kg^−1^ FW)**
PYTZ	0.74 ± 0.016^b^	0.25 ± 0.027^b^	0.99 ± 0.023^b^	2.94 ± 0.029^a^	0.28 ± 0.007^b^
	1.36 ± 0.024^a^	0.46 ± 0.021^a^	1.82 ± 0.008^a^	2.93 ± 0.017^a^	0.46 ± 0.019^a^
RX	0.95 ± 0.013^b^	0.36 ± 0.015^b^	1.31 ± 0.029^b^	2.65 ± 0.041^a^	0.39 ± 0.015^b^
	1.62 ± 0.032^a^	0.72 ± 0.031^a^	2.33 ± 0.037^a^	2.26 ± 0.022^b^	0.62 ± 0.023^a^

PYTZ, Pingyang Tezao; RX, Ruixue. Superscript letters a and b indicate significant differences between before and after fertilization of the same variety (*p* <0.05). The 1st and 2nd line of the same variety is the control and experimental group separately.

## Discussion

The results of our comprehensive nutrient analysis of soils in Rizhao and Shouzhao, and elemental analysis and morphological comparisons of tea leaves grown in these regions, showed that high-pH Shouguang soil was not deficient in N, Zn, Cu, or Mn (Table 5), but that uptake of these elements in tea plants grown in Shouguang was significantly lower than in Rizhao, indicated by the lower levels of these elements in the leaves (Table 4). These deficiencies led to the destruction of leaf chloroplast structure (Table 3) and hindered chloroplast synthesis (Figure 1), resulting in yellowing disease and albino symptoms in the leaves. N deficiency was the likely cause of yellowing in the lower adult leaves of tea plants; the content of Mn and Cu in the new yellowing leaves was significantly lower than the normal range for tea leaves, which may have been the cause (3, 17). Zhou et al. (18) reported that Mn deficiency in tea plants mainly manifests as changes in leaf color, which is as follows: first, the greenish-yellowing of newly developed young leaves, which extends from the leaf green to the front half of the leaves, slight downward-bending, and leaf tips and edges with a scorched appearance. It has been reported that the lack of Zn in young leaves causes darkening, brownspot formation, chartreuse color in mature leaves, destruction of cellular structures, reductions in photosynthesis and chlorophyll contents, and lack of Cu leaf color loss of green into yellow. In severe cases, all leaves turn yellow (2, 14). These previous findings are consistent with our experimental results (Figure 1; Table 2). N, Cu, Mn, and Zn contents in tea leaves significantly increased with the application of foliar fertilizer; chlorophyll content was also significantly increased, yellowing diseases and albinism were not present, and leaves were clearly green, indicating that the plants were healthy.

If the effective Ca content in the soil exceeds 500 mg kg^−1^, it is not suitable for tea plant cultivation (19). Under high-pH conditions, excessive Ca treatment was previously shown to cause damage to tea roots (19). Excessive levels of Ca in tea leaves can destroy the membrane structure of the photosynthetic system, resulting in obstruction of the electron transport chain and reductions in the light energy utilization efficiency of tea leaves, thereby affecting tea growth and pigment content (15, 20). Some studies have shown that excess Ca reduces the uptake of Zn and K by the tea plants (21, 22). The effective Ca level in Shouguang soil was 6543.3 mg kg^−1^, which was 9.90 times higher than that of Rizhao soil. Excessive Ca in high-pH soil likely damaged the chloroplast structure, hindering pigment synthesis and inhibiting Zn and K uptake by tea plants. This likely contributed to the yellowing and albinism we observed in tea plant leaves.

While high-pH soil may not lack the elements required by healthy tea plants, high pH reduces the ability of tea plants to absorb them. The application of acidic fertilizers in crop cultivation, therefore, lowers the soil pH and activates these elements, facilitating absorption. The commonly used acidifiers include sulfur, aluminum sulfate, and ferrous sulfate. In addition, it has been reported that Zn and Mn are not easily absorbed by tea plants in high-pH soil, and their deficiency can be corrected by foliar fertilizer application, while soil application is not effective (23). Foliar application of Cu in the form of inorganic salts, oxides, or chelates may be used to rapidly correct Cu deficiency in soil-grown plants (24). Accordingly, the use of foliar spray fertilizer may directly and effectively supplement these elements in the leaves, facilitating the prevention and control of yellowing diseases and albinism in tea leaves, enhancing the quality of the leaves with clearly observable effects, as shown in our study.

## Conclusion

The leaves of tea plants grown in high-pH areas (Shouguang) showed yellowing diseases and albino, with serious damage to the cellular ultrastructure, reduced pigment content, and reduced main quality components; the analysis was combined with the elemental contents of the tea leaves and the soil. The main reason for the yellowing diseases and albino of tea leaves is that the high-pH soil hinders the uptake of N, Zn, Cu, and Mn by tea plants, causing the chloroplasts to suffer and the synthesis of chlorophyll to be hindered; in addition, the high content of Ca in high-pH areas is also an important reason for the poor growth of tea plants. The high-pH soil hinders the uptake of P and K by tea plants, causing a decline in the main quality components such as tea polyphenols and amino acids. The yellowing diseases and albino of tea plants can be controlled and the main quality components of tea can be improved by spraying a compound foliar fertilizer of N, P, K, Zn, Cu, and Mn.

## Data availability statement

The original contributions presented in the study are included in the article/supplementary material, further inquiries can be directed to the corresponding author.

## Author contributions

C-LL conceived and designed the study, conducted the experiments, and analyzed the data. JL, L-XZ, and Z-KW presented the methodology and investigation. H-MX and JX collected the literature and translated the manuscript. All authors contributed to the article and approved the submitted version.
